# Dr. George C. Jarvis

**Published:** 1901-06

**Authors:** 


### OBITUARY.

Dr. George C. Jarvis, of Hartford, Conn., died of pneumonia at his home in that city May 7, 1901, aged 67 years. He graduated in arts at Trinity College in 1855, and received his doctorate degree at the University of the City of New York in 1860. He was commissioned as surgeon of the 7th Regiment, Connecticut Volunteers during the civil war and served with credit in that capacity. He was consulting surgeon at the Hartford Hospital and medical director of the Connecticut Mutual Life Insurance Company.

